# Prevalence and Predictors of Somatic Symptoms among Child and Adolescents with Probable Posttraumatic Stress Disorder: A Cross-Sectional Study Conducted in 21 Primary and Secondary Schools after an Earthquake

**DOI:** 10.1371/journal.pone.0137101

**Published:** 2015-09-01

**Authors:** Ye Zhang, Jun Zhang, Shenyue Zhu, Changhui Du, Wei Zhang

**Affiliations:** 1 Mental Health Center, West China Hospital, Sichuan University, Chengdu, Sichuan, China; 2 Education Supervision Department, Baoxing County Education Bureau, Yaan, Sichuan, China; 3 Science and Education Information Department, Chengdu Center of Disease Control, Chengdu, Sichuan, China; National Center of Neurology and Psychiatry, JAPAN

## Abstract

**Purpose:**

To explore the prevalence rates and predictors of somatic symptoms among child and adolescent survivors with probable posttraumatic stress disorder (PTSD) after an earthquake.

**Methods:**

A total of 3053 students from 21 primary and secondary schools in Baoxing County were administered the Patient Health Questionnaire-13 (PHQ-13), a short version of PHQ-15 without the two items about sexuality and menstruation, the Children's Revised Impact of Event Scale (CRIES), and the self-made Earthquake-Related Experience Questionnaire 3 months after the Lushan earthquake.

**Results:**

Among child and adolescent survivors, the prevalence rates of all somatic symptoms were higher in the probable PTSD group compared with the controls. The most frequent somatic symptoms were trouble sleeping (83.2%), feeling tired or having low energy (74.4%), stomach pain (63.2%), dizziness (58.1%), and headache (57.7%) in the probable PTSD group. Older age, having lost family members, having witnessed someone get seriously injured, and having witnessed someone get buried were predictors for somatic symptoms among child and adolescent survivors with probable PTSD.

**Conclusions:**

Somatic symptoms among child and adolescent earthquake survivors with probable PTSD in schools were common, and predictors of these somatic symptoms were identified. These findings may help those providing psychological health programs to find the child and adolescent students with probable PTSD who are at high risk of somatic symptoms in schools after an earthquake in China.

## Introduction

On 20 April 2013, an earthquake registering 7.0 on the Richter scale occurred in Sichuan Province, China. The epicenter was located in Lushan County, which was also affected by the Wenchuan Earthquake in 2008. There were only five years between these two catastrophic disasters. The Lushan earthquake resulted in 196 deaths; furthermore, there were 21 missing, at least 11,470 injured and more than 968 seriously injured [1], causing psychological changes among survivors [2].

Survivors affected by disaster have more opportunities to develop posttraumatic stress disorder (PTSD) [3] and are more likely to have comorbid somatic symptoms [4–6]. Previous studies found that PTSD was possibly associated with a higher frequency of somatic symptoms compared with other psychiatric disorders [7, 8]; there was a preponderance of neurological, cardiovascular, musculoskeletal, respiratory and gastrointestinal disorders, as well as diabetes, chronic pain and sleep disorders [9]. Considering their potential impact on the course of PTSD and the functional disability associated with it, comorbid somatic symptoms are a critical concern in PTSD [7].

A number of studies have explored the somatic conditions of survivors after various kinds of disasters. For example, 6 months following deployment, the most frequent somatic symptoms reported among infantry soldiers [10] were sleep problems (32.8%), musculoskeletal pain (32.7%), fatigue (32.3%), and back pain (28.1%). In Rwanda, 14 years after the genocide, the most common somatic symptoms were back pain (74.1%) and headache (72.5%) among the survivors with PTSD [11]. As for the child and adolescent survivors who are in their early period of psychological development, they are more easily impacted by traumatic events and more vulnerable to develop severe psychological problems [12] or somatic symptoms [13] after disaster. One of the few studies conducted in children and adolescents after the Wenchuan earthquake reported the severity of somatic complaints in the respiratory system, cardiovascular system, nervous system, digestive system, and urogenital system, but not the prevalence rates in child and adolescents with PTSD [14]. Therefore, evidence regarding the prevalence rates of somatic symptoms in children and adolescents with PTSD should be added to the literature.

Earthquake-related experiences were found to be associated with PTSD in a number of studies [15–17]. These experiences included having witnessed someone get buried [16], having witnessed someone get injured [16], and losing family members [18]. Nonetheless, little is known about whether earthquake-related experiences could make contributions to the somatic symptoms among child and adolescent survivors suffering from PTSD after an earthquake.

Based on the literature, we hypothesized that child and adolescent survivors with probable PTSD displayed high prevalence rates of somatic symptoms and that several earthquake-related experiences (i.e., having witnessed someone almost get hit, having witnessed someone get seriously injured, having witnessed someone get buried, losing family members, etc.) could contribute to the somatic symptoms. The aims of the present study were to explore the prevalence rates of somatic symptoms and identify the predictors of somatic symptoms among children and adolescents with probable PTSD after the Lushan earthquake.

## Methods

### Participants and Procedure

Three months after the Lushan earthquake, the survey was conducted in Baoxing County, which was one of the severely damaged counties during the earthquake. It was selected for two reasons: (a) there were 21 primary and secondary schools in this county, which could satisfy the requirement for the large sample in the present study, and (b) the manager of the Education Bureau of Baoxing County and the schools’ principals and teachers in these schools were aware that it is important to perform an investigation regarding the psychosomatic conditions which could benefit the psychosocial intervention in child and adolescent students, and they were willing to provide assistance for this study. In the present study, we did not provide any type of treatment for the probable PTSD students. However, we have returned these results to the teachers and principals in these schools, and they will provide some useful information and more supports to the probable PTSD students.

**Fig 1:**
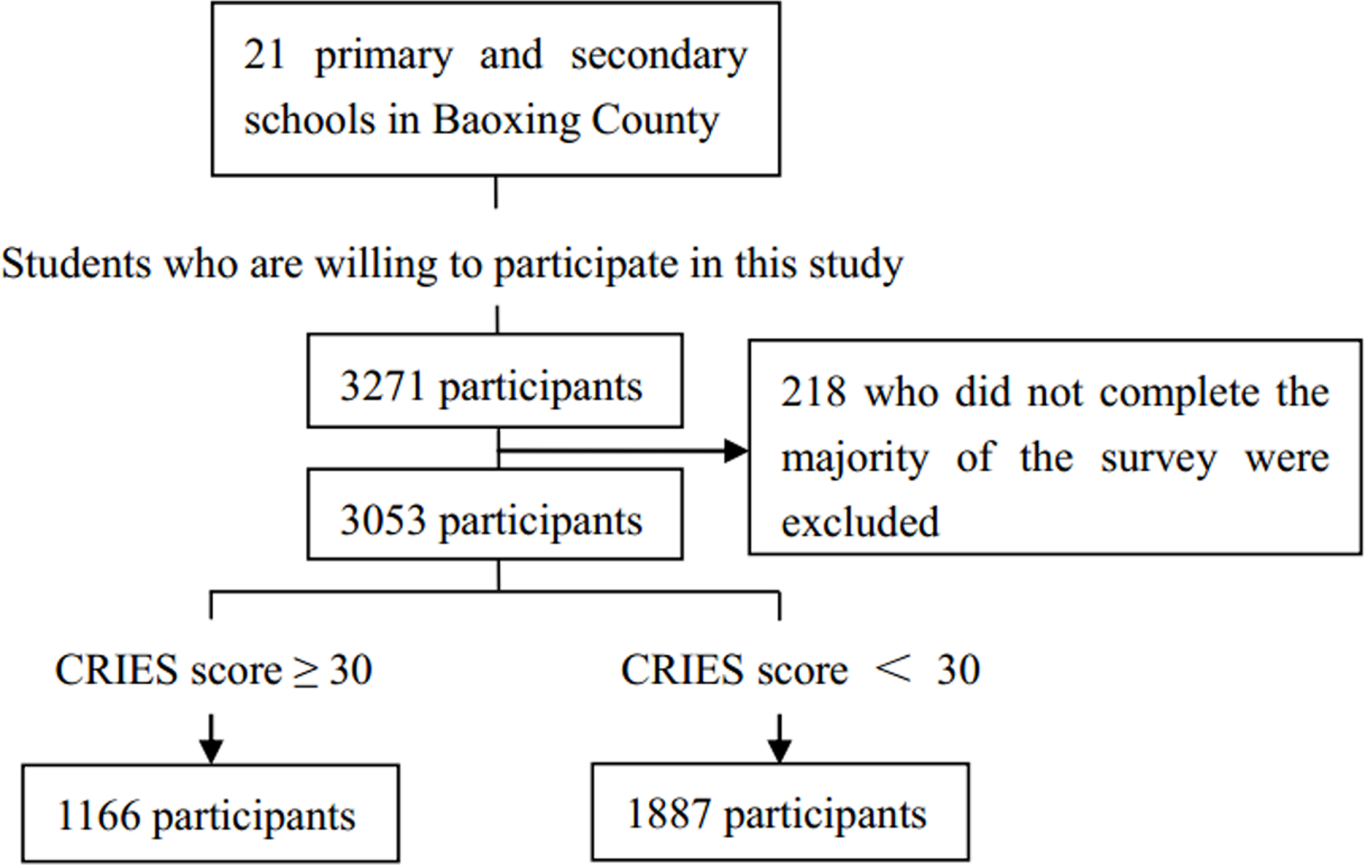
Flow diagram of the participants.

The sampling strategy is shown in Fig 1. The data were collected at the end of July 2013, more than 3 months after the Lushan earthquake. The investigation process was completed within 7 days to control for the effects of time. A sample of 3271 participants was recruited from the 21 primary and secondary schools in Baoxing County. The inclusion criterion was willingness to participate, and the exclusion criteria were refusal to participate and failure to complete the majority of the survey. Of the possible 3271 participants, 218 were excluded because the respondents did not complete the majority of the survey, yielding a response rate of 93.3%. Finally, data for 3053 respondents were complete and suitable for analysis.

This study was approved by the Research Ethics Committee of the West China Hospital of Sichuan University, the Education Bureau of the Baoxing County, and the Department of Health of Sichuan Province. Written informed consent was obtained from school principals and teachers. In China, research projects do not need parental consent if local education authorities, such as the county Bureau of Education and school administrators, approve it as a service to the students [19]. The present study belonged to that category; thus, written informed consent from parents was not required [19]. Before conducting this investigation, investigators gave child and adolescent survivors a description of the procedures and informed them that they could join this study voluntarily and that they had a right to withdraw from this study. Written informed consent was obtained from each student in the questionnaire. Under the supervision of trained individuals with a master’s degree in psychology, participants completed the following questionnaires in their classrooms: the Patient Health Questionnaire-13(PHQ-13) scale, the Children’s Revised Impact of Event Scale (CRIES), and the self-made Earthquake-Related Experience Questionnaire.

### Measures

The evaluation tools of this study included two parts. The first part was to assess demographic characteristics (i.e., age, ethnicity and gender) and earthquake-related experiences. Earthquake-related experiences were measured by a self-reported questionnaire, which included the following questions: (1) Did you almost get hit by destroyed objects in the Lushan earthquake? (2) Did you get hit and injured in the Lushan earthquake? (3) Did you witness someone almost get hit in the Lushan earthquake? (4) Did you witness someone get seriously injured in the Lushan earthquake? (5) Did you witness someone get buried in the Lushan earthquake? (6) Have you lost family members in the Lushan earthquake? (7) Did you feel anxious about not seeing family members in the Lushan earthquake? (8) Do you have peace of mind currently? All these questions were coded into yes/no items.

The second part was to evaluate PTSD and somatic symptoms. The PHQ-15 is a 15-item self-report scale to measure somatic symptoms. Participants need to evaluate the extent of the somatic symptoms on a 3-point scale ranging from “not bothered at all” (value = 0) to “bothered a lot” (value = 2). The total score is 28 for men and 30 for women (one item each is about menstrual problems and sexuality) [20]. Since participants in this study are students in primary and secondary schools, we used the PHQ-13, a short version of the PHQ-15 without the two items about menstruation and sexuality, to screen the somatic symptoms of participants. The total score of the PHQ-13 in this study is 26 for both boys and girls. In the current study, each somatic symptom was dichotomized using “bothered a lot or bothered a little”/“not bothered at all” as the cutoff. We re-coded the variables regarding the somatic symptoms to fulfill the aim to estimate the prevalence rates of somatic symptoms. We defined the individuals suffering from somatic symptoms as individuals who reported “bothered a lot or bothered a little” in the PHQ-13 scale and individuals not suffering from somatic symptoms as individuals who reported “not bothered at all” in the PHQ-13 scale. The Chinese version of the PHQ-15 shows satisfactory internal consistency and reliability in the general population of China [21]. The Cronbach’s alpha of the PHQ-13 scale was 0.86 in the present study.

The Children's Revised Impact of Event Scale (CRIES) is a 13-item self-report scale to measure children’s traumatic symptoms including intrusion, avoidance, and hyperarousal aspects. Participants are asked to finish the extent of traumatic symptoms on a 4-point scale, ranging from “not at all” (value = 0) to “often” (value = 5). The total score is 65. A high score is an indicator of a high level of traumatic symptoms. We use probable PTSD in this study because of the gold-standard structured diagnostic interview are not performed in our sample. Previous studies have demonstrated that a score of 30 and above is the most effective cutoff score to screen cases of probable PTSD [22, 23]. In the present study, we defined the probable PTSD group as individuals with CRIES score ≥ 30 and the control group as individuals with CRIES score < 30.The CRIES has been shown to have good validity and reliability in assessing adolescents’ traumatic symptoms in China [24]. The Cronbach’s alpha of the scale was 0.89 in the present study.

### Statistical Analysis

Descriptive statistics were computed for categorical variables. A Chi-square test was used to compare the differences in the prevalence rates of somatic symptoms between the probable PTSD group and the control group. Spearman and Pearson correlation analyses were used to detect the relationship between demography, earthquake-related experiences, and somatic symptoms in the probable PTSD group, no PTSD group and the whole sample. We used the total score of the PHQ-13 scale as the dependent variable. Age, ethnicity, gender, earthquake-related experiences and the CRIES score were included as candidate variables in the stepwise regression analyses to detect the predictors of somatic symptoms in the probable PTSD group, no PTSD group and the whole sample. Statistical analyses and calculations were performed using the Statistical Package for the Social Sciences (SPSS) for Windows (version 19.0). A two-tailed *P* <0.05 was considered statistically significant.

## Results

### Demographic Characteristics

As shown in Table 1, of the 3053 participants, 1545 (50.6%) were female, and 2674 (87.6%) were Han. The mean age was 12.01±2.62 years (range, 8 to 19 years). There were more female in the probable PTSD group than in the control group. Earthquake-related experiences of the participants are also presented. There were more participants with the experiences of almost getting hit by destroyed objects, having witnessed someone almost get hit, having witnessed someone get seriously injured, feeling anxious about not seeing family members, and not having peace of mind currently in the probable PTSD group compared with those in the control group.

**Table 1:** Description of study population.

			No. (%)^a^			
		All	Probable	Control		
	Variable	participants	PTSD group	group		
Characteristics	Assignment	(N = 3053)	(N = 1166)	(N = 1887)	*χ* ^*2*^ */t*	*p*
Almost getting hit by	Yes	641 (21.0)	292 (25.0)	349 (18.5)	17.138	< .001
destroyed objects	No	2242 (73.4)	819 (70.2)	1423 (75.4)		
Getting hit and injured	Yes	139 (4.6)	54 (4.6)	85 (4.5)	.009	.926
	No	2759 (90.4)	1061 (91.0)	1698 (90.0)		
Having witnessed someone	Yes	1039 (34.0)	453 (38.9)	586 (31.1)	15.137	< .001
almost get hit	No	1915 (62.7)	695 (59.6)	1220 (64.7)		
Having witnessed someone	Yes	630 (20.6)	296 (25.4)	334 (17.7)	22.739	< .001
get seriously injured	No	2323 (76.1)	849 (72.8)	1474 (78.1)		
Having witnessed someone	Yes	183 (6.0)	79 (6.8)	104 (5.5)	1.593	.207
get buried	No	2768 (90.7)	1065 (91.3)	1703 (90.2)		
Lost family members	Yes	375 (12.3)	161 (13.8)	214 (11.3)	3.182	.074
	No	2578 (84.4)	983 (84.3)	1595 (84.5)		
Feeling anxious about not	Yes	1675 (54.9)	699 (59.9)	976 (51.7)	14.206	< .001
seeing family members	No	1283 (42.0)	448 (38.4)	835 (44.3)		
Not having peace of mind	Yes	944 (30.9)	407 (34.9)	573 (28.5)	6.702	.010
currently	No	2004 (65.6)	734 (63.0)	1270 (67.3)		
Gender	Male	1502 (49.2)	494 (42.4)	1008 (53.4)	34.981	< .001
	Female	1545 (50.6)	669 (57.4)	876 (46.4)		
Ethnicity	Han	2674 (87.6)	1025 (87.9)	1649 (87.4)	.103	.748
	Minorities	371 (12.2)	139 (11.9)	232 (12.3)		
Age (*Mean*±*SD*)		12.01±2.62	12.28±2.57	11.85±2.63	4.361	< .001

^a^ Percentages do not always correspond to the total N due to cases with insufficient data.

### Prevalence Rates of Somatic Symptoms

As shown in Table 2, the prevalence rates of all somatic symptoms (feeling tired or having low energy; trouble sleeping; headache; dizziness; stomach pain; back pain; chest pain; pain in the arms, legs or joints; fainting spells; feeling your heart pound or race; shortness of breath; constipation, loose bowels, or diarrhea; and nausea, gas, or indigestion) in the probable PTSD group were significantly higher compared with the control group (*p* < .001). The most frequent somatic symptoms were trouble sleeping, feeling tired or having low energy, stomach pain, dizziness, and headache.

**Table 2:** Prevalence rates of somatic symptoms in the probable PTSD group and control group.

	No. (%)			
	Probable PTSD group^a^	Control group^a^		
Somatic symptoms	(N = 1166)	(N = 1887)	*χ* ^*2*^	*p*
Stomach pain			218.898	< .001
Not bothered at all	421 (36.8)	1204 (64.5)		
Bothered a little or bothered a lot	723 (63.2)	663 (35.5)		
Back pain			114.895	< .001
Not bothered at all	795 (70.3)	1604 (86.4)		
Bothered a little or bothered a lot	336 (29.7)	253 (13.6)		
Chest pain			121.558	< .001
Not bothered at all	868 (76.9)	1687 (91.4)		
Bothered a little or bothered a lot	261 (23.1)	159 (8.6)		
Pain in arms, legs, or joints			175.690	< .001
Not bothered at all	659 (58.7)	1504 (81.0)		
Bothered a little or bothered a lot	463 (41.2)	351 (19.0)		
Headache			170.712	< .001
Not bothered at all	479 (42.2)	1236 (66.6)		
Bothered a little or bothered a lot	655 (57.7)	620 (33.4)		
Dizziness			165.519	< .001
Not bothered at all	475 (41.9)	1224 (65.9)		
Bothered a little or bothered a lot	658 (58.1)	632 (34.1)		
Fainting spells			22.562	< .001
Not bothered at all	988 (88.0)	1722 (93.1)		
Bothered a little or bothered a lot	135 (12.0)	128 (6.9)		
Feeling your heart pound or race			197.654	< .001
Not bothered at all	597 (52.9)	1440 (77.6)		
Bothered a little or bothered a lot	531 (47.0)	415 (22.4)		
Shortness of breath			172.889	< .001
Not bothered at all	770 (68.3)	1627 (87.9)		
Bothered a little or bothered a lot	358 (31.7)	223 (12.1)		
Constipation, loose bowels, or diarrhea			136.552	< .001
Not bothered at all	757 (67.1)	1576 (85.3)		
Bothered a little or bothered a lot	371 (32.9)	272 (14.7)		
Nausea, gas, or indigestion			265.323	< .001
Not bothered at all	589 (52.1)	1488 (80.3)		
Bothered a little or bothered a lot	542 (47.9)	364 (19.7)		
Feeling tired or having low energy			324.641	< .001
Not bothered at all	289 (25.6)	1100 (59.6)		
Bothered a little or bothered a lot	840 (74.4)	747 (40.4)		
Trouble sleeping			438.994	< .001
Not bothered at all	191 (16.8)	1030 (55.5)		
Bothered a little or bothered a lot	949 (83.2)	826 (44.5)		

^a^ The total N does not always correspond to 1166 for the probable PTSD group and 1887 for the control group due to cases with insufficient data.

### Relationship between Main Variables and Somatic Symptoms in the Probable PTSD Group

As shown in Table 3, in the probable PTSD group, age was positively and significantly associated with somatic symptoms (r = 0.192). Gender and ethnicity were not associated with somatic symptoms. Almost all the earthquake-related experiences, except for getting hit and injured and having witnessed someone almost get hit, were positively and significantly associated with somatic symptoms, with correlation coefficients ranging from 0.062 to 0.131. The results of the correlation analysis conducted in the no PTSD group and the whole sample are also shown in Table 3.

**Table 3:** Relationship between main variables and total PHQ-13 score.

			Total PHQ-13 score			
	All participants		Probable PTSD		Control group	
	(N = 3053)		(N = 1166)		(N = 1887)	
Variables	r	*p*	r	*p*	r	*p*
Gender	.081	< .001	-.035	.26	.088	< .001
Age	.173	< .001	.192	< .001	.127	< .001
Ethnicity	-.001	.964	-.008	.80	-.003	.902
Almost getting hit by destroyed objects	.084	< .001	.068	.031	.045	.066
Getting hit and injured	.021	.287	.045	.16	.011	.663
Having witnessed someone almost get hit	.114	< .001	.039	.21	.097	< .001
Having witnessed someone get seriously injured	.118	< .001	.127	< .001	.066	.007
Having witnessed someone get buried	.050	.008	.131	< .001	-.008	.735
Lost family members	.050	.008	.070	.025	.021	.374
Feeling anxious about not seeing family members	.107	< .001	.080	.009	.087	< .001
Not having peace of mind currently	.045	.018	.062	.048	-.001	.961

Gender was coded as 1 = male and 2 = female; Ethnicity was coded as 1 = Han and 2 = minority; each earthquake-related experience was coded as 1 = no and 2 = yes.

### Predictors of Somatic Symptoms for the Probable PTSD Individuals

Multiple regression analyses were conducted to explore the effects of demographic variables (i.e., age, ethnicity and gender) and earthquake-related experiences on somatic symptoms. As shown in Table 4, after controlling for the effects of PTSD symptoms, older age, having lost family members, having witnessed someone get seriously injured, and having witnessed someone get buried were predictors for somatic symptoms. Together, these variables accounted for 4.3% of the variance of somatic symptoms. The results of the multiple regression analyses conducted in the no PTSD group and the whole sample are also shown in Table 4.

**Table 4:** Predictors of somatic symptoms in the subgroups and all participants.

				Adjusted	
Final models	B	BE	Beta	R^2^	△R^2^
**Probable PTSD (N = 1166)**				.152	
CRIES score	.165	.016	.309***		.113
Age	.272	.058	.140***		.023
Lost family members	1.181	.402	.086**		.009
Having witnessed someone get seriously injured	.798	.324	.073*		.006
Having witnessed someone get buried	1.338	.579	.069*		.005
**No PTSD (N = 1887)**				.121	
CRIES score	.119	.009	.311***		.102
Having witnessed someone almost get hit	.781	.171	.107***		.012
Age	.103	.031	.077**		.007
Having witnessed someone get seriously injured	.418	.207	.047*		.002
**All participants(N = 3053)**				.285	
CRIES score	.138	.005	.493***		.266
Age	.170	.029	.098***		.011
Lost family members	.725	.219	.055**		.004
Having witnessed someone get seriously injured	.555	.182	.051**		.002
Having witnessed someone almost get hit	.430	.155	.047**		.002
Having witnessed someone get buried	.693	.311	.037*		.001

*p < .05

**p < .01

***p < .001; Gender was coded as 1 = male and 2 = female; Ethnicity was coded as 1 = Han and 2 = minority; each earthquake-related experience was coded as 1 = no and 2 = yes.

## Discussion

To our knowledge, the present study is the first research to investigate the prevalence rates and the predictors of somatic symptoms among child and adolescent survivors with probable PTSD. Our results demonstrated that the prevalence rates of all somatic symptoms among children and adolescents with probable PTSD in schools were higher compared with the control group, and the most frequent somatic symptoms were trouble sleeping, feeling tired or having low energy, stomach pain, dizziness, and headache. There were more participants with earthquake-related experiences in the probable PTSD group compared with those in the control group. The results also indicated that, after controlling for the effects of PTSD symptoms, child and adolescent survivors with probable PTSD in schools were more likely to have comorbid somatic symptoms if they were at an older age, had witnessed someone get seriously injured, had lost family members, or had witnessed someone get buried.

### Prevalence of Various Somatic Symptoms Among Individuals with Probable PTSD

In the present study, the prevalence rates of all somatic symptoms in the probable PTSD group were higher compared with the control group, which is consistent with previous studies done in adults. Munyandamutsaet al. [11] found all somatic symptoms, including headache, back pain, abdominal pain, genital pain, sexual difficulties, fainting, hiccups, loss of speech, and hearing loss, among the survivors with PTSD were more frequent compared with survivors without PTSD 14 years after the genocide in Rwanda. Gupta et al. [9] reported a higher frequency of similar medical conditions in individuals suffering from PTSD compared with the trauma-alone group without PTSD.

The most frequent somatic symptom among children and adolescents with probable PTSD was trouble sleeping (83.2%), which is in accordance with previous studies. Up to 87% of PTSD individuals reported subjective sleep disturbances [25] that were resistant to first-line treatments, independently exacerbated daytime symptoms, and contributed to poor clinical outcomes, including poorer perceived physical health and a higher rate of suicidality [26]. It is necessary to pay more attention to the sleep problems of child and adolescent survivors with PTSD to get more satisfactory clinical outcomes.

The prevalence of feeling tired or having low energy was 74.4% in the probable PTSD group. Fatigue in PTSD was also reported in previous studies. Spinhoven et al. [27] found an increased level of fatigue in residents (45.4%) and rescuers (20.6%) after involvement in a disaster. Nater et al. [28] reported that chronic fatigue syndrome (CFS) was associated with PTSD, with a>25% comorbid rate with PTSD [26]; furthermore, PTSD symptoms could predict CFS [29].

High prevalence rates of pain symptoms, including headache (57.7%), back pain (29.7%), chest pain (23.1%), and pain in the arms, legs, or joints (41.2%), existed in the probable PTSD group in the current study. In accordance with previous studies, PTSD could be comorbid with a wide range of chronic pain syndromes such as episodic migraine, headache [30] and other atypical and typical pain symptoms [31–34]. In Rwanda, 14 years after the genocide, the prevalence rates of several pain symptoms including headache (72.5%), genital pain (30.3%), abdominal pain (44.2%), and back pain (74.1%) were reported among the survivors with PTSD [11]. It is worth noting that PTSD comorbid with pain symptoms may impede the patient’s ability to benefit from the treatments of pain [35] and PTSD [36].

A total of 47% of children and adolescents with probable PTSD had the symptom of feeling your heart pound or race, supporting previous studies in which individuals suffering from PTSD displayed greater heart rates in the emergency room than those without PTSD [37], and PTSD was related to electrocardiographic evidence of atrioventricular conduction defects and unexplained supraventricular tachycardia [9, 38].

The prevalence of shortness of breath was 31.7% among children and adolescents with probable PTSD, which is similar to previous studies. Approximately 5 to 6 years after the terrorist attacks on 11 September 2001, a large burden of PTSD and asthma resulting from acute, intense exposure and prolonged exposures was reported [39]. Although the pathogenetic mechanisms of asthma and PTSD were different, their co-occurrence was often observed [39], and PTSD could significantly predict asthma symptoms [40]. Therefore, it is not difficult to understand the frequent occurrence of shortness of breath among children and adolescents with PTSD.

As reported in previous studies among survivors with PTSD, we also found the existence of dizziness and fainting spells with a prevalence of 58.1% and 12.0%, respectively, in the probable PTSD group. Fourteen years after the genocide in Rwanda, the prevalence of fainting was 9.2% [11], and chronic dizziness could be considered a feature of anxiety disorders including PTSD [9, 41].

As to the gastrointestinal system in the present study, stomach pain (63.2%), constipation, loose bowels, or diarrhea (32.9%), and nausea, gas, or indigestion (47.9%) were detected in the probable PTSD group, suggesting that gastrointestinal symptoms were common among child and adolescent survivors with PTSD after an earthquake. This is similar to the findings of prior studies that psychological trauma was related to the high frequency of functional gastrointestinal disorders [42, 43]. Many other studies also have found obvious associations between PTSD and gastroesophageal reflux symptoms, idiopathic constipation, functional dyspepsia and irritable bowel syndrome [44–48]. It is important to choose an effective intervention to mitigate the gastrointestinal symptoms of child and adolescent survivors with PTSD in the early period after earthquakes.

### Predictors of Somatic Symptoms Among Children and Adolescents with Probable PTSD

The substantial number of somatic symptoms reported by adolescents, with girls reporting higher levels than boys, is atypical finding for health surveys in the general population [49–51]. A longitudinal study of urban adolescents also indicated that girls displayed higher levels of somatic anxiety and somatic complaints compared with boys [52]. In the present study, female gender was not a predictor for somatic symptoms in the whole sample and in the subgroups which was inconsistent with previous studies. One possible explanation was that we investigated a trauma sample in the present study as opposed to the general population or no trauma sample in previous studies, which should be further investigated in future.

Just as the finding that older age was a predictor for somatic symptoms among child and adolescents in the two subgroups and in all participants, Sun et al. [14] found that older children and adolescents had relatively more serious somatic symptoms compared with the younger children and adolescents after the Wenchuan earthquake and attributed this situation to the excessive attention, pessimistic expectations and negative cognitive perception of traumatic events by older children and adolescents [14].

In the present study, after controlling for the effects of PTSD symptoms, several earthquake-related experiences were predictors for somatic symptoms among children and adolescents, especially having witnessed someone get seriously injured had a significant predictive effect in both the probable PTSD group and the control group. This is consistent with a conclusion that somatic symptoms are associated with a stress event and psychological trauma [53, 54]. A recent meta-analysis revealed that individuals who were exposed to a traumatic event were more likely to develop functional somatic symptoms with a 2.7 times higher risk compared with those without traumatic experiences [55]. A recent review also indicated that a trauma event or psychological trauma was associated with somatic symptoms among individuals with PTSD after disasters [9]. These predictors, explored with the regression model for somatic symptoms in the current study, could help to screen and diagnose somatic symptoms among child and adolescent survivors with PTSD after an earthquake.

There are several limitations to the study. Firstly, using the self-report questionnaires to estimate PTSD and somatic symptoms might overestimate the prevalence rates of PTSD and somatic symptoms. Secondly, we did not take steps to recognize whether a somatic symptom resulted from some organic diseases, which might generate bias in the results. Thirdly, the survey was performed when the students were in school, and the self-made questionnaire did not include the item regarding whether the students had the experience of living in temporary shelters. Because of this selection bias, our results should be interpreted with caution.

In summary, this study indicated that somatic symptoms among child and adolescent survivors with probable PTSD in schools were commonly seen. Older age, having lost family members, having witnessed someone get seriously injured, and having witnessed someone get buried could predict somatic symptoms among child and adolescents survivors with probable PTSD in schools. More importantly, these findings may help those providing psychological health programs to find the child and adolescent students with probable PTSD who are at high risk of somatic symptoms in schools after an earthquake in China.

## Supporting Information

S1 Dataset (SAV)
